# Current Management Strategies in Breast Cancer by Targeting Key Altered Molecular Players

**DOI:** 10.3389/fonc.2016.00045

**Published:** 2016-03-01

**Authors:** Shazia Ali, Neelima Mondal, Hani Choudhry, Mahmood Rasool, Peter N. Pushparaj, Mohammad A. Khan, Maryam Mahfooz, Ghufrana A. Sami, Jummanah Jarullah, Ashraf Ali, Mohammad S. Jamal

**Affiliations:** ^1^School of Life Science, Jawaharlal Nehru University, New Delhi, India; ^2^Department of Biochemistry, Faculty of Science, Center of Innovation in Personalized Medicine, King Fahd Center for Medical Research, King Abdulaziz University, Jeddah, Saudi Arabia; ^3^Center of Excellence in Genomic Medicine Research, King Abdulaziz University, Jeddah, Saudi Arabia; ^4^National Institute of Biologicals, Noida, India; ^5^Department of Computer Science, Jamia Millia Islamia, New Delhi, India; ^6^Department of Biotechnology, Jamia Millia Islamia, New Delhi, India; ^7^King Fahd Medical Research Center, King Abdulaziz University, Jeddah, Saudi Arabia

**Keywords:** breast cancer, chemoprevention, aromatase inhibitors, epidermal growth factor receptor, estrogen receptor, biomarker

## Abstract

Breast cancer is the second largest disease affecting women worldwide. It remains the most frequently reported and leading cause of death among women in both developed and developing countries. Tamoxifen and raloxifene are commonly used selective estrogen receptor modulators for treatment of breast cancer in women with high risk, although resistance occurs by tamoxifen after 5 years of therapy and both drugs cause uterine cancer and thromboembolic events. Aromatase inhibitors (AIs) are one of the optional modes used for breast cancer treatment. The combination of AIs along with tamoxifen can also be beneficial. Various therapeutic agents from different sources are being studied, which further need to be improved for potential outcome. For this, clinical trials based on large number of patients with optimal dose and lesser side effects have to be more in practice. Despite the clinical trials going on, there is need of better molecular models, which can identify high risk population, new agents with better benefit having less side effects, and improved biomarkers for treating breast cancer.

## Introduction

Breast cancer is the second leading cause of death in the female population worldwide. Each year, 200,000 new cases of invasive breast cancer are diagnosed (1). Breast cancer comprises 22.9% of all cancers worldwide (2). The survival rates and prognosis for breast cancer are mostly dependent on the type of cancer, stages, treatment, and the ethnicity and location of the patient. High survival rates have been observed in breast cancer cases of western world as compared to developing countries where survival rates are less. Out of 10 women, 8 or more (85%) in England having breast cancer survive for at least 5 years. There are a number of factors responsible for causing breast cancer. Dietary factors, such as high-fat diet, alcohol intake, smoking, obesity, higher levels of cholesterol, and iodine deficiency have high risk of cancer occurrence. In females, lack of breastfeeding and childbearing make them more susceptible to disease. About 9% cancer cases can be prevented by changing diet and body weight, e.g., Japanese women have less rate of breast cancer than Americans but when they shift their place, risk increases. European Prospective Investigation into Cancer has shown that menopausal women having more fat are at high risk, high carbohydrate diet also increase the risk of breast cancer. People who ate fish, dairy products, fiber, fruits, vegetables, flavonoids, and antioxidants have low risk (3). Asian women who eat more soy foods have low risk of breast cancer. In a cohort study in USA, 10 g of pure alcohol a day is limited for women and 20 g for men (4). Smoking is another factor, which increases the risk of breast cancer, especially those who smoke at an early age in their life have risk as high as 35–50% (5, 6). There are other risk factors involved in cancer occurrence, such as chemicals biphenyls, solvents of organic nature, hydrocarbons, pesticide use, radiation, and hazardous shift work.

Genetics has an important role in breast cancer. In about <5% of cases, breast–ovarian cancer syndrome occurs by inheritance including women having BRCA1 and BRCA2 mutation. The 90% of total genetics account to these mutations with a breast cancer risk of 60–80% affected cases. BRCA1 mutations predispose women to breast and ovarian cancers. BRCA1 in breast cancer has higher aneuploidy number than tumors, which do not have mutations in BRCA1 (7, 8). The next section discusses the molecular alterations during breast cancer, how various therapies and agents can prevent breast cancer, and how these agents can be modulated with better efficacy and positive outcome in treatment of the disease.

## Molecular Alterations in Breast Cancer

### Cell Cycle Deregulation in Cancer

The growth and differentiation of a cell is controlled by a regulated cell cycle. If there is uncontrolled proliferation of cells, it leads to cancer. This results in abnormal functioning of tissues, organ system in an organism. The cell cycle has checkpoints, which check the entry of cell from one phase to another phase and whether the functions are completed in each phase or not. These checkpoints are controlled by genes, which help in stimulating or inhibiting the cell division. These are proto-oncogenes and tumor suppressor genes. The cell cycle-dependent kinases are regulated by cyclins present in a cell and are required for cell division. They are regulated by CDK inhibitors, such as INK4 proteins and p21 and p27 of Cip and Kip family. The deregulation of CDKs results in uncontrolled proliferation and genomic defects in DNA repair mechanisms and DNA damage checkpoints and chromosomal instability. Mitotic checkpoints and DNA damage alterations cause increase in function of CDK, which leads to initiation of tumor. The cell cycle regulatory signals are not controlled as in a normal process of cell cycle. This leads to deregulated cell cycle, mutations, and genetic abnormalities (9).

## Role of Epidermal Growth Factor Receptor in Breast Cancer

Epidermal growth factor receptor is from ErbB class of tyrosine kinase cell surface-based receptor including HER2, neu, or ErbB2 (10). The epidermal growth factor receptor family comprises four cell surface receptors, EGFR are HER1, HER2 or neu, HER3, and HER4 types. The binding of growth factor to receptor activates its dimerization with other members of ErbB, such as HER2, and phosphorylation occurs. This makes binding sites available for cytosolic proteins containing Src homology 2 (SH2) domains and leads to stimulation of downstream factors activating mitogen-activated protein kinase pathway, which causes cell to re-enter S-phase of cell cycle, consequently resulting in cell proliferation (11). The ligands of EGFR include transforming growth factor α (TGF-α), amphiregulin, epigen, betacellulin, heparin binding factor, and epiregulin (12). EGFR amplification, increase in copy number of gene occurs in 15–30% of breast cancer cases. This results in poor prognosis in patients and decreased survival rate (13–15).

## Role of H2AX (H2A Histone Family, Member X) in Breast Cancer

H2AFX is the genes coding histone H2A protein. The H2A, H2B, H3, and H4 are histone proteins present in two copies and form a histone octamer. The DNA is wrapped around these histones and forms nucleosome. DNA damage response is activated upon DNA damage in cells. Double-strand breaks activate phosphorylation of histone variant H2AX, which repairs DNA by activating proteins at damaged chromatin and at checkpoints arresting cell cycle. γ-H2AX helps to develop cancer therapies (9, 16, 17). It also occurs due to other factors, such as shortened telomeres and hypoxia (18). It has been found that in triple negative, in BRCA1 and p53-mutated breast cancer γ-H2AX level is more (19), and in triple negative, the chance of errors are more in DNA damage repair pathway (20). DNA damage response signaling is marked by γH2AX in familial breast tumors and in ER, PR, ERBB2-triple negative breast carcinoma.

## Role of Poly ADP Ribose Polymerase in Breast Cancer

PARP1 is a protein which repairs single-strand breaks in the DNA. PARP1 is also involved in differentiation, proliferation, and tumor transformation (21). It decreases the ATP level of a cell upon repairing DNA damage which results in lysis and cell death (necrosis). It also causes programmed cell death by production of PARP, which activates mitochondria to release apoptosis inducing factor. PARP inactivation occurs by enzymes caspases or cathepsins, which cause cleavage of PARP. The cleavage fragments tell which cell death pathway is activated. Double-strand DNA breaks are repaired by homologous recombination repair pathway having BRCA1 and BRCA2 proteins. If they are mutated, it causes errors in repair of DNA resulting in breast cancer. PARP1 protein repairs single-strand breaks; if it is not repaired, it causes double-strand breaks. There are drugs which are used as PARP inhibitors. PARP inhibitors are used in cancer cells with mutated BRCA1 and BRCA2 proteins. Cancer cells are sensitive to PARP inhibitors. Iniparib, olaparib, and rucaparib are some of the PARP inhibitors used for breast cancer (22).

## Role of p53 in Breast Cancer

Tumor suppressor protein, p53 is encoded by the TP53 gene in humans, which functions to inhibit cell proliferation to regulate cell cycle. It is known as the guardian of the genome as it maintains the cellular stability by preventing genetic mutation. In normal cells, low level of p53 is maintained by continuous degradation via Mdm-2, which is an ubiquitin ligase. The p53 activation occurs when there is a cellular stresses, such as DNA damage (23), phosphorylation of p53 occurs due to which it is no longer degraded by Mdm-2 and hence accumulates. Post-translational modifications stimulate the protein for DNA binding, transactivating downstream effector genes which regulate the action of tumor suppressor p53. Upon DNA damage or cellular stress, p53 is known to activate apoptosis or cell cycle arrest. It has major role in cancer as it maintains genomic stability, anti-antigenic effects, manages tumor inflammation, and immune response. TP53 is mutated mostly in 50% of all human cancers and in 20–30% of breast cancers with more than 15,000 different mutations. About 30% of the TP53 mutations are because of genetic changes in breast cancer. It varies with tumor subtype (24, 25). The mutant p53 affects various other proteins in a cell leading to metastasis.

## Reactive Oxygen Species in Breast Cancer

Reactive oxygen species (ROS) contain oxygen reacting molecules and regulate cell signaling and homeostasis (26). It is generated and eliminated at the same time in normal process, balanced by scavenging system. Due to environmental stress, such as UV or heat exposure, ROS levels increase, damaging cell causing carboxylation of cellular proteins and peroxidation of lipids resulting in carcinogenesis. ROS level can decide the difference between tumor and non-tumor cells. In cancer cells, stress causes increase in metabolism, mitochondrial dysfunction and in levels of ROS (27, 28). ROS in cancer cell stimulates several transcription factors, such as NF-κB, AP-1, HIF-1α, ATF 3, and STAT3, leading to expression of protein for cell growth, defense, and survivability including cell proliferation, invasion leading to metastasis (29). ROS is useful for survival of cancer cells in moderate level and in excessive level, kills cancer cells. If there is increased level of oxidative stress in mitochondria, cytochrome *c* is released, apoptosis occurs, and stimulation of caspases leads to cell death resulting in stimulation of c-Jun N-terminal kinases (JNKs). JNK phosphorylates and downregulates the Bcl-2 and Bcl-XL, anti-apoptotic proteins. This leads to damage to mitochondrial membrane (30). The molecular alterations in breast cancer are triggered by cascade of reaction in a cell and are dependent on one another. ROS are produced by metabolic activities of cell and cytotoxic drugs. DNA damage by ROS activates PARP-1, which in turn organizes DNA repair by modifications of proteins, such as histones and helicases. PARP leads to decrease in NAD, ATP level in cell resulting in cell death and necrosis regulated by p53, which induced apoptosis and senescence upon exposure to ROS (31).

## Biomarkers as a Tool for Breast Cancer

The upcoming studies are in practice based on whole genome arrays collection from patients, such as next-generation sequencing (NGS). Ion torrent sequencing is used for cancer DNA sequencing, which is less expensive (32). DNA and RNA sequencing is done from tumors based on exomes or candidate genes, which identifies somatic mutations and malignant transformation in significantly mutated genes SMG (33). In cancer genome atlas data, genes mutated in luminal A, B, and basal such as breast cancer are listed, TP53, PIK3CA, MAP3K1, and PTEN, which are used as a therapeutic tool in triple negative breast cancer. Somatic mutation in cancer based on copy number, aberrations, nucleotide substitutions, and on subsets in breast cancer BRCA1/2 are found by dGene, DGIdb HER2, and ESR1 estrogen receptor (ER) gene mutations for finding a drug or kinase inhibitor. Patient-derived xenograft is used now as a genomic model to avoid genetic drift, which should have larger sample size (34). The microarray data and the samples of biological use can be saved in an independent bio bank to identify new prognostic or predictive biomarkers and drug targets. PARP inhibitors are new drugs given as a single therapy or in combination with various agents of DNA damage such as radiation therapy. These inhibitors have fewer side effects and are used to treat aggressive cancers, such as cancers involving hereditary BRCA1/2 and triple negative breast and ovarian cancer. PARP inhibitor biomarkers, such as olaparib for patients with BRCA1/2-mutant tumors, ovarian, and colorectal cancers; iniparib for breast and lung cancers; rucaparib for breast and ovarian cancer; and veliparib for melanoma and breast cancer, are used. The crosstalk of DNA repair pathways occur, which requires combination of DNA repair biomarkers. The discovery of biomarkers has led to develop effective cure with beneficial clinical outcome (35).

## Therapeutic Approaches for Breast Cancer

### Chemotherapy

Chemotherapy is used in ER-negative breast cancer (ER^−^) occurring in stages 2–4. The chemotherapeutic drugs are given in combinations for about a period of 3–6 months. One combination is known as AC, which is combination of cyclophosphamide with doxorubicin (adriamycin) (36). Another class of drugs are taxanes, in which docetaxel and taxotere are used in combinations known as CAT (37). CMF is the combination of cyclophosphamide, methotrexate, and fluorouracil. Cisplatin or methotrexate as single agents or lipoic acid and hydroxycitrate combined together or used alone is not as effective as lipoic acid, hydroxycitrate, and cisplatin or methotrexate combined together. It targets metabolic pathway in cancer associated with classical chemotherapy (38).

### Monoclonal Antibodies

HER2 is a tyrosine kinase-based cell receptor of epidermal growth factor group, which is overexpressed in some of the breast cancer cells. In breast cancer, the disease recurs and has less prognosis in about 25–30% cases where HER2 gene and its protein is overexpressed (39). As an adjuvant therapy, monoclonal antibody trastuzumab (Herceptin) is used to treat HER2-positive breast cancers which are in stages 1–3 and has benefited about 87% of patients for 5-year survival (40). Trastuzumab binds to HER2 overexpressing cancer cells, thereby blocks effectively growth of breast cancer. Trastuzumab is expensive and causes serious side effects. About 2% of patients undergoing treatment with this drug suffer significant heart damage. It is used for patients having metastasis as a combination with chemotherapy or as monotherapy, lapatinib is used first in combination with letrozole or HER2, ER (+) breast cancer patients as well for HER2 (+) in combination with capecitabine (41).

### Hormonal Therapy

Hormones are naturally occurring substances in the body whose function is to work as chemical messengers. There are various approaches, which have been used to treat hormone responsive breast cancer.

#### Blocking Ovarian Function

Ovaries are the primary source of estrogen in premenopausal women. By eliminating or suppressing ovarian function, estrogen levels are reduced. Ovarian function when inhibited is called as ovarian ablation. It is permanent in which ovaries are removed called as oophorectomy or by treating with surgery and radiation. Another method is suppression of ovaries with drugs, which is not a permanent treatment and is called as gonadotropin (GnRH) or luteinizing hormone releasing hormone (LH-RH) agonists. They inhibit the ovaries to produce estrogen and prevents stimulus from the pituitary gland. The U.S. Food and Drug Administration has approved drugs for ovarian ablation, which are goserelin and leuprolide (42).

#### Blocking Estrogen Production

In breast cells, estrogen helps in cell growth. Estrogen is made in the ovaries and tissues by aromatase enzyme. Estrogen binds to ER on the surface of cell. ERs reside on the surface, in cytoplasm, and in nucleus. When estrogen molecules are not present, these receptors are inactive but once the estrogen molecule enters cell, the estrogen binds to its receptors causing change in conformation of receptor. ER complex binds at their DNA-binding sites called as estrogen response elements. As a neoadjuvant therapy, drugs are used to inhibit the function of an enzyme called aromatase. They are given in postmenopausal women. The drug is used in combination in order to suppress ovarian function, as ovaries produce more aromatase and cannot be blocked by inhibitors in premenopausal women (43). Anastrozole and letrozole are the aromatase inhibitors (AIs) both of which inhibit aromatase for short period, while exemestane permanently inhibits the enzyme.

#### Blocking Estrogen’s Effects

There are various drugs which intervene with estrogen action to activate the growth of breast cancer cells. SERMs are agents modulating function of estrogen in tissues. ERs are activated or inhibited in different tissues. The function of SERMs varies in different tissues depending on chemical structure of selective estrogen receptor modulator (SERM) as an antagonist in breast tissue and agonist in other tissues, such as bone and uterus. Tamoxifen in breast functions as an antagonist and in uterus as agonist. It has been found that the amount of co-activator 1 (SRC-1 and NCOA1) is in excess amount in uterus as compared to breast. Raloxifene in both acts as an antagonist. It has been found that raloxifene potentially enlists co-repressor proteins and in uterus acts as an antagonist. The three main drugs that act SERMs are tamoxifen (Nolvadex), raloxifene (Evista), and toremifene (Fareston) (44). Other antiestrogen drugs: fulvestrant (Faslodex) upon binding to the ER acts as an estrogen antagonist as SERMs, but it acts as antiestrogen, which upon binding to ER, causes destruction of receptor and same does not occur in SERMs. Fulvestrant is used with other antiestrogens in combination and with AIs such as anastrozole, letrozole, and exemestane in ER postmenopausal women having metastasis.

### Aromatase Inhibitors

**Figure 1:**
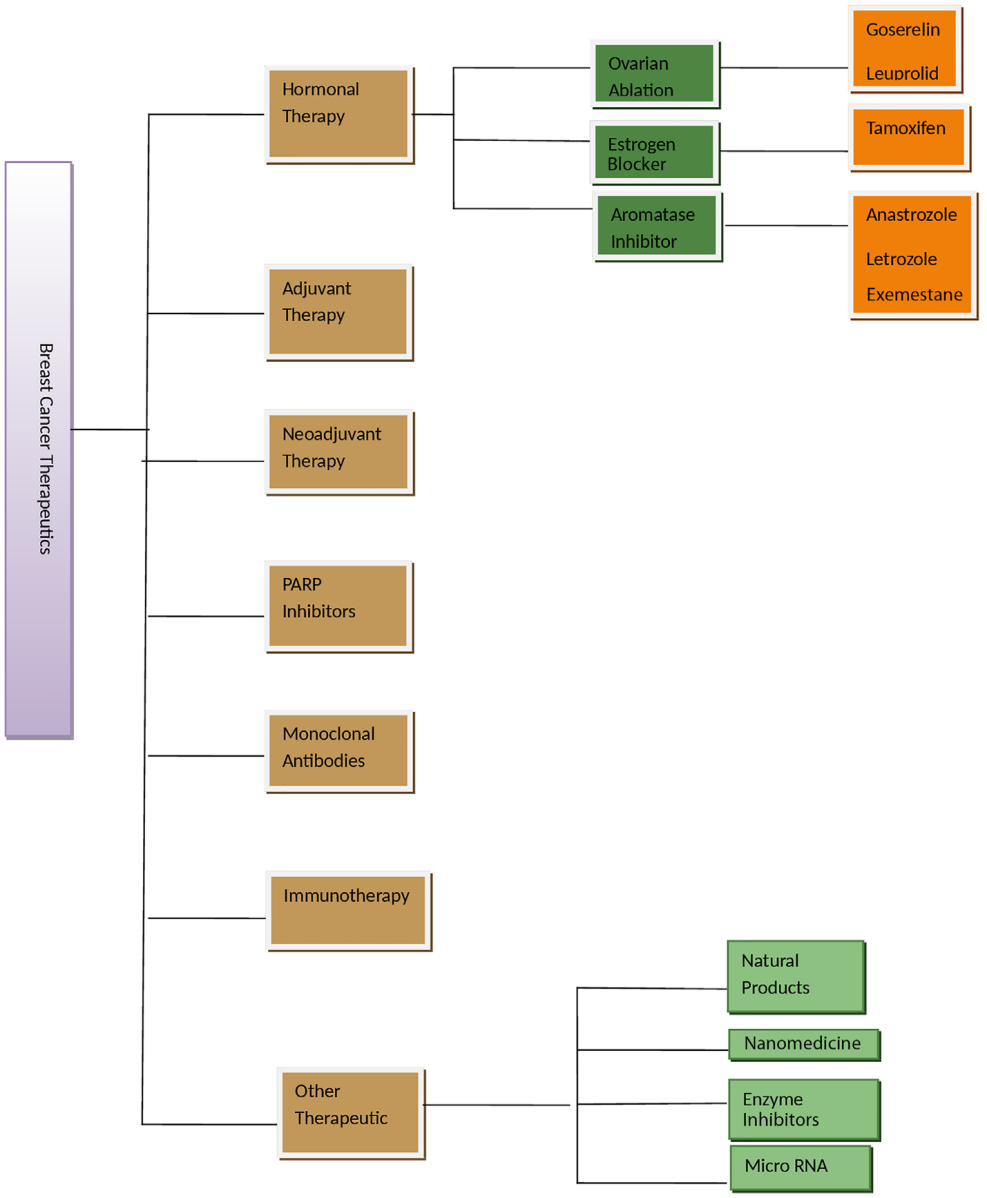
**Figure showing various therapeutic options for breast cancer management**. Adjuvant therapy is given after surgery or main treatment and may include radiation or chemical therapy. Neoadjuvant therapy is given before main treatment. Other therapeutics is in experimental phase.

Aromatase inhibitors are used to inhibit endogenous formation of estrogen from androgens. These act by inhibiting the function of the aromatase enzyme complex, which catalyzes this conversion (45). The activity of enzyme is inhibited by binding to it, leading to the formation of inactive enzyme, which is incapable of binding on its actual site of androgen substrate. Aromatase forms estrogen in ovaries and in several tissues of the body. First AI, formestane, was used to compare tamoxifen treatment and its effect in clinical trials and had same effect as of tamoxifen. The other AIs including anastrozole, letrozole, and exemestane (46, 47) had increased efficiency when studied in combination in various clinical trials than tamoxifen alone. The AIs, anastrozole and letrozole, are given as first-line drugs to postmenopausal hormone-sensitive women in the advanced stages of breast cancer (48). AIs are not given to premenopausal women, as they are not able to inhibit the enzyme as it is formed more in ovaries of these women. The drugs are effective when given in combination with other drugs that inhibits ovarian function in these women. There are few side effects of AI, such as blood clots, stroke, bone loss, and heart problem. The main modalities of treating breast cancer, which are described in Figure 1, can be improved by pharmacological studies of therapies and drugs. In therapeutic agents, preclinical studies need to be validated in clinical studies with factors, such as large sample number, less toxicity, and combinatorial studies. Hormonal therapy also requires more sample number, less toxicity, and optimal dose. Monoclonal antibodies and non-hormonal therapy are more toxic, which can be replaced by use of biomarkers where we need to check efficacy, toxicity, and avoid intra-heterogeneity in breast cancer patients. Other therapeutic agents in use are suberoylanilide hydroxamic acid (SAHA), second generation compound, and a histone deacetylase inhibitor. SAHA reduces growth, causes cell cycle arrest, and apoptosis in breast cancer cells of human (49).

## Agents Commonly Used for Breast Cancer Control

Natural compounds have been used in treatment of breast cancer, for example, luteolin in herbs, such as thyme, parsley, and vegetables, such as celery and broccolis, are used in women who have taken hormone replacement therapy. Luteolin reduces vessels feeding cancer cells. In another study, chemical nutrients were tested in broccoli, grapes, apples, tofu, and turmeric root (50). The compounds curcumin, isoflavone, indole-3-carbinol, c-phycocyanin, resveratrol, and quercetin used in combination were effective in reducing breast cancer cell growth by 80% and there was no effect on control cells (51). Some of natural compounds are listed in Table 1.

**Table 1:** **(A)** Selective natural compounds used in breast cancer therapy; **(B)** selective preclinical and clinical studies of novel agents for breast cancer prevention.

(A) SELECTIVE NATURAL COMPOUNDS USED IN BREAST CANCER THERAPY				
Compound	Source	Studies	Effect	Reference
**Flavonoid**				
Honokiol	*Magnolia officinalis* and *Magnolia grandiflora*	ER^+^ MCF-7, MDA-MB-231 ER^−^ breast cancer	It arrests cell cycle, leads to apoptosis in cancer cells, and acts as antioxidant	(52)
Magnolol	*Magnolia officinalis* and *Magnolia obovata*	MDA-MB-231	It causes cell cycle arrest, apoptosis, and acts as antiproliferative agent	(53)
**Sesquiterpenes**				
Costunolide	*Inula helenium*, *Saussurea lappa*, and *Magnolia grandiflora*	MCF-7, MDA-MB-231	It arrests cell cycle leads to apoptosis in cancer cells, and acts as antioxidant	(54)
Parthenolide	*Tanacetum parthenium*, *Tanacetum vulgare*, *Centaurea ainetensis*, *Tanacetum larvatum*, and *Helianthus annuus*	MCF-7, MDA-MB-231	It arrests cell cycle, leads to apoptosis in cancer cells, and acts as antioxidant. Cytotoxic	(55)
**Diterpenoids**				
Pseudolaric acid B	*Pseudolarix kaempferi*	MCF-7, MDA-MB-231	It arrests cell cycle, leads to apoptosis in cancer cells, and acts as antioxidant	(56)
Oridonin	*Isodon rubescens*	MCF-7, MDA-MB-231	It arrests cell cycle, leads to apoptosis in cancer cells, and acts as antioxidant. Autophagic agent	(57)
**Polyphenolic**				
Wedelolactone	*Eclipta alba*, *Wedelia calandulaceae*, and *Wedelia chinensis*	MDA-MB-231,468	It arrests cell cycle, leads to apoptosis in cancer cells, and acts as antioxidant	(58)
**Alkaloids**				
Evodiamine	*Evodia rutaecarpa*	MCF-7	It arrests cell cycle, leads to apoptosis in cancer cells, and acts as antioxidant, antimetastatic, and anticarcinogenesis	(59)
**(B) SELECTIVE PRECLINICAL AND CLINICAL STUDIES OF NOVEL AGENTS FOR BREAST CANCER PREVENTION**				
**Drugs/agents**	**Studies: *in vitro*, *in vivo*, trials**	**Effect on breast cancer**	**Source**	**Reference**
Beta-lactam	MCF-7 and MDA MB-231 breast cancer cells, xenograft mouse model	It inhibits proliferation of breast cancer cells and tumor growth in mouse model. Beta lactamase linked affinity reagents based on cancer cell fusion peptides can be used directly in targeted enzyme prodrug development in cancer	β-lactam ring are group of antibiotics such as penicillins, carbapenems, and monobactams	(60–63)
Triphenylethylenes	ER-positive MCF-7 and the ER-negative breast cancer cell line T47D, BALB/c athymic mice	It is used for breast cancer treatment, examples are tamoxifen, idoxifene, and toremifene. Tamoxifen is used in ER^+^ breast cancer, its dose 20 mg/day is optimized in ongoing clinical trials to reduce toxicity	Non-steroidal antiestrogens	(64)
Letrozole	MCF-7 breast cancer cells, MCF-7Ca tumor xenograft models and BALB/c athymic nude mice BIG 1-98 study group clinical trial compared letrozole and tamoxifen drug in breast cancer patients	It is used in local or advanced breast cancer having hormone receptor positive. It is used in combination with tamoxifen with improved overall survival	Non-steroidal aromatase inhibitor	(65, 66)
Anastrozole	Murine breast cancer cells (4T1) in female BALB/c mice ATAC clinical trial compared anastrozole and tamoxifen for treatment of breast cancer	The combination trial of ATAC showed that it has more efficiency and less side effects than tamoxifen and can be used as initial treatment for postmenopausal women with ER^+^ breast cancer	Non-steroidal aromatase inhibitor	(67)
Cyclosporin A	Multidrug-resistant human breast cancer cells MCF-7-adriamycin-resistant (AdrR), female athymic nude BALB/c mice	It lowers levels of glucosylceramide in multidrug-resistant cells which are given tamoxifen. It functions as a chemoresponsive agent. Pharmacokinetics of docetaxel in combination of CsA showed active and safer use for treating advanced breast cancer in Phase II study	It is an immunosuppressant drug	(68, 69)
Verapamil	BALB/c mouse murine breast cancer cells (4T1-R)	It inhibits multi drug resistance rendering cells sensitive to chemotherapy at an optimal concentration of 6 and 1–2M	It functions as an L-type calcium blocker from group of phenylalkylamine	(70, 71)
Suramin	MDA-MB-231 cells, xenografted human, athymic mice	The drug binds to TGF, EGFR, FGF, PDGF, and IGF causing impaired growth of cell and is used for breast cancer treatment. In combination with paclitaxel, it is effective and non-cytotoxic in metastatic breast cancer at 10 and 50 μmol/l concentrations in phase I and II trials	It functions as an antagonist of P2 receptors which are ATP-stimulated G protein-coupled receptors	(72, 73)
Flaxseed	MCF-7 breast cancer cells, ovariectomized mice, nude mice	It inhibits the growth of human estrogen-dependent breast cancer in athymic mice, and it enhances the inhibitory effect of tamoxifen. Dietary flaxseed reduces tumor growth in breast cancer and is less expensive and available	Flaxseed (FS) is rich in mammalian lignan precursors and α-linolenic acid, which have anticancer effects	(74, 75)
Plumbagin	Human breast cancer cell MDA-MB-23, female BALB/c mice	Plumbagin reduces cancer cell growth and osteoclast formation in the bone of mice	It was isolated form plant plumbago	(76)

*EGFR, epidermal growth factor; TGF, transforming growth factors alpha and beta; FGF, fibroblast growth factors; PDGF, platelet-derived growth factor; IGF, insulin-like growth factors; ATAC, anastrozole, tamoxifen combination trial*.

There are various other agents which are used in combination with tamoxifen in breast cancer. Some of the agents are mentioned in Table 1.

## Conclusion

Antiestrogen and estrogen therapies are developed with better outcome by finding out markers which can differentiate between Phases I and II breast cancer to overcome resistivity of ­different sets of drug. Anticancerous drugs are given in high doses, assumed on the basis of population studies. The pharmacological approach which decides the nature and mode of action of chemotherapy is in need of standardization of dose and response to a drug with better efficiency and less toxicity. A set of new biomarkers for breast cancer are needed to improve risk factors. Clinical trials need to be done in population having high risk with large samples and longer follow-up. The side effects and dose is needed to evaluate for different set of population having diversity. The chemoprevention and other therapeutic agents can be improved by keeping in mind above factors by physicians, oncologists, and patients. There is a need of molecular models for identification of high risk factors to identify new agents with more benefit ratio and less toxicity as a drug development program. Various approaches, such as improved clinical trials, combinatorial approach of different drugs, gene expression studies, and pharmacokinetics, will benefit right patients with right kind of drugs.

## Author Contributions

SA: draft and writing, design, concept, editing; NM: design, concept, editing and approved; HC: draft and writing; MR: editing; PNP: editing; MAK: editing; MM: writing; GAS: writing; JJ: editing; AA: writing; MSJ: design, concept, editing, and approved.

## Conflict of Interest Statement

The authors declare that the research was conducted in the absence of any commercial or financial relationships that could be construed as a potential conflict of interest.

## Abbreviations

AIs, aromatase inhibitors; AP-1, activator protein-1; EGFR, epidermal growth factor receptor; ER, estrogen receptor; H2AX, H2A histone family, member X; HIF, hypoxia-inducible factor-1α; NF-κB, nuclear factor kappa-light-chain-enhancer of activated B cells; PARP, poly-ADP ribose polymerase; ROS, reactive oxygen species; SAHA, suberoylanilide hydroxamic acid; SERM, selective estrogen receptor modulators; STAT3, signal transducer and activator of transcription 3; TGF, transforming growth factor.
